# Health, Functional and Nutritional Determinants of Falls Experienced in the Previous Year—A Cross-Sectional Study in a Geriatric Ward

**DOI:** 10.3390/ijerph17134768

**Published:** 2020-07-02

**Authors:** Lukasz Magnuszewski, Marta Swietek, Agnieszka Kasiukiewicz, Bartlomiej Kuprjanowicz, Jan Baczek, Zyta Beata Wojszel

**Affiliations:** 1 Doctoral Studies, Department of Geriatrics, Faculty of Health Sciences, Medical University of Bialystok, 15-471 Białystok, Poland; magnuszewskilukas@gmail.com (L.M.); marta.swietek@umb.edu.pl (M.S.); janbaczek@gmail.com (J.B.); 2 Department of Geriatrics, Hospital of the Ministry of Interior and Administration in Bialystok, 15-471 Bialystok, Poland; akasiukiewicz@o2.pl (A.K.); barti.bial@wp.pl (B.K.); 3 Department of Geriatrics, Medical University of Bialystok, 15-471 Bialystok, Poland

**Keywords:** fall risk factors, fall detection, fall epidemiology, geriatrics, older patients

## Abstract

Falls are a serious health problem in older adults. A limited number of studies assessed their multifactorial nature in geriatric ward patients. The aim of this study is to explore health, functional and nutritional correlates of experiencing fall(s) in the previous year by older inpatients. A cross-sectional study of patients admitted to the department of geriatrics was conducted. A “faller status” was defined based on positive history of falls in the previous 12 months. Health, functional and nutritional factors associated with falls were evaluated, and multivariable logistic regression analysis models were built. A total of 358 patients (median age 82 (IQR 76–86) years, 77.9% women) were recruited, 43.9% of whom reported falls. The “fallers” presented with a significantly higher number of chronic diseases, higher prevalence of Parkinson’s disease, peripheral arterial disease, chronic osteoarthritis, more frequently reported urinary incontinence and were dependent on others for daily living activities. They had significantly worse results for the assessment of gait, balance and frailty status. The Mini Nutritional Assessment-Short Form scores and the mean value of serum albumin were significantly lower in the fallers’ group. Parkinson’s disease (OR = 2.82, CI—1.07–7.45; *p* = 0.04) and osteoarthritis (OR = 2.08, CI—1.02–4.23; *p* = 0.04) were the main variables for the outcome prediction, according to the direct multivariable logistic regression analysis. Our findings suggest that Parkinson’s disease and osteoarthritis are the main predictors independently associated with a history of falls in patients admitted to the geriatric ward, although the influence of some factors may be underestimated due to the tendency of not taking the history of falls in very frail, functionally dependent and bedridden individuals.

## 1. Introduction

Falls are a major health problem in older adults. A high percentage of the geriatric population experienced a fall each year [1,2]. Falls are connected with high morbidity, increased odds for hospitalization caused by serious injuries, increased risk of mortality, fear of falling (post-fall syndrome), and lead to lower quality of life [3].

Many intrinsic and extrinsic factors can contribute to or cause falls. The intrinsic factors include gait and balance disorders, cognitive deterioration, abnormal nutritional status, orthostatic hypotension and different medical conditions such as Parkinson’s disease, stroke and its consequences or chronic osteoarthritis, among others [4,5,6,7,8,9]. The extrinsic factors include maladjustment of the living environment to the disability of an older person, polypharmacy, taking certain groups of a drug for treatments (such as psychotropics, diuretics or antihypertensive medications) [10,11,12]. Furthermore, sarcopenia—defined as low skeletal muscle mass, strength, and physical performance has been pointed recently as a potentially modifiable risk factor of falls in older people [13]. Both groups of factors—internal and external—are interrelated.

The aim of the study was to assess clinical health, functional and nutritional characteristics associated with the history of pre-hospitalization falls in geriatric inpatients, taking into account the multifactorial nature of this “geriatric syndrome” [14]. To the authors’ knowledge, a limited number of studies examined determinants of faller status in patients hospitalized in a geriatric ward from the multifactorial perspective, based on the comprehensive geriatric assessment [15]. The problem of falls was most often assessed in community-dwelling older people and the risk factors taken into account were relatively few [2]. Studies carried out in hospital departments concentrated, for instance, on the risk of falls in patients who have fallen in hospital, in the context of the possibility of preventing falls in inpatients [16,17], selectively analyzed some risk factors for falls, such as the type of medication taken [18] or assessed the impact of the risk of falls on prognosis after discharge from hospital [19].

## 2. Materials and Methods

Our study is a secondary analysis of the data collected across the cross-sectional study on frailty and multimorbidity in patients of the department of geriatrics (Hospital of the Ministry of Interior and Administration in Bialystok, Poland) [20]. All consecutive patients, admitted for the first time to the department of geriatrics on the turn of 2014 and 2015, took part in it. The department of geriatrics is a subacute care ward; patients are admitted primarily electively. Most often, they are burdened with chronic diseases and various disabilities. One of the main goals of their stay in the ward is to conduct a comprehensive geriatric assessment and to create a long-term care plan. For this reason, in most cases, it is not possible to indicate one specific reason for hospitalization. Patient examinations were carried out by permanent employees of the ward: geriatricians and specialists in internal medicine, nurses specialized in geriatrics or long-term care, a clinical neuropsychologist and a physiotherapist.

The analysis included patients who—alone or represented by a guardian—answered the question about the occurrence of falls in the last 12 months. Participants who confirmed such incidents were treated as having a “faller” status, and those who denied them as “non-fallers”.

### 2.1. Patient Characteristics

Sociodemographic characteristics included age, gender, place of residence (urban/rural). History taking included information on 15 chronic diseases (peripheral arterial disease, ischemic heart disease, chronic cardiac failure, myocardial infarction, hypertension, atrial fibrillation, history of transient ischemic attack (TIA) or stroke, chronic obstructive pulmonary disease, diabetes or prediabetes, neoplasm, dementia, parkinsonism, chronic osteoarthritis, osteoporosis and chronic renal disease), drug for treatments taken before hospitalization that could influence the risk of falls (antihypertensive drugs: angiotensin converting enzyme inhibitors (ACE-Is)/ angiotensin II receptor blockers (ARBs), beta-blockers, calcium channel blockers, diuretics, α1-blockers; digoxin; lipid lowering drugs; antidepressants: selective serotonin reuptake inhibitors (SSRI); neuroleptics: in general and quetiapine separately; pro-cognitive medications: memantine, acetylcholinesterase inhibitors (AChE-Is), benzodiazepines (BDA); non-steroidal anti-inflammatory drugs (NSAIDs)) and hospitalizations in the last 12 months. Information obtained from the patient was verified by an interview with his or her guardian, by a thorough clinical examination, and by a review of all of the patient’s medical records available.

### 2.2. Measurements

The functional profile of a patient was created based on the results of comprehensive geriatric assessment routinely conducted at the department. Risk of recurrent falls was assessed with previously validated tests—the Performance-Oriented Mobility Assessment (POMA) [21] and the Timed Up-and-Go test (TUG) [22]. Walking speed was measured during the 4.6 m walk from standing position in usual gait speed (the fastest time of 2 trials was used). Hand grip strength was measured in the dominant hand according to the Southampton protocol, using a hand-held hydraulic dynamometer DHD-1 (SAEHAN, Changwon, Korea) [23]. The ability to perform basic activities of daily living was assessed with the Barthel index [24] and instrumental activities of daily living (IADL) with 6 items of the Duke Older American Resources and Services (OARS) I-ADL [25]. Cognitive abilities were evaluated using the Abbreviated Mental Test Score (AMTS) [26] and the possibility of depression with the 15-item Geriatric Depression Scale (GDS) [27]. The diagnosis of dementia at discharge was made on the basis of the neuropsychologist’s examination during the patient’s hospital stay. The 7-item Canadian Study of Health and Aging Clinical Frailty Scale (CSHA-CFS) was used to asses frailty [28]. Risk of malnutrition was determined with the Mini Nutritional Assessment Short form (MNA-SF) [29]. Body mass ndex (BMI) was measured according to the standard procedure.

Systolic and diastolic blood pressure was measured at admission. Orthostatic hypotension occurrence was assessed with active standing test performed by a physiotherapist in the morning on the second day of the patient’s hospital stay. After a resting period of 10 min, blood pressure was measured electronically (Automatic Philips IntelliVue MP5 Monitor, Amsterdam, The Netherlands) on the non-dominant arm with the patient lying down. The patient was then asked to stand up, and the blood pressure was measured again during the first and the third minute after standing [30]. If the patient was not able to stand, the measurement was made in a sitting position.

Data on vitamin B12, serum sodium, creatinine, albumin, and hemoglobin levels was extracted from patients’ medical records. Renal function was assessed with glomerular filtration rate—GFR, counted using the Chronic Kidney Disease Epidemiology Collaboration (CKD-EPI) formula [31].

### 2.3. Study Parameters

Multimorbidity was defined as the co-occurrence of 5 or more diseases of the 15 listed above. Polypharmacy was defined as 5 or more drugs taken. Severe frailty was defined as a CFS score of 6 or 7 [28]. Orthostatic hypotension was defined by a drop in blood pressure of at least 20 mmHg for systolic blood pressure and at least 10 mmHg for diastolic blood pressure within 3 min of standing up. Chronic kidney disease, i.e., stages 3, 4 and 5 CKD according to Kidney Disease Outcome Quality Initiative (KDOQI), was diagnosed if the GFR was < 60 mL/min/1.73 m^2^. Anemia was diagnosed if the hemoglobin level was below 8.7-mmol/ L in men and below 7.5-mmol/L in women. Patients with BMI ≥ 30 kg/m^2^ were classified as obese, and with BMI < 24 kg/m^2^ as being at risk for malnutrition. Malnutrition was also suspected if the MNA-SF score was below 8. Dynapenia (or probable sarcopenia) was derived from the handgrip strength and diagnosed in men if grip strength was lower than 27 kg and in women if it was lower than 16 kg [32]. It was classified as severe based on physical performance; if low grip strength was accompanied by gait speed equal or lower than 0.8 m/s, and/or TUG equal or higher than 20 s, the patient was diagnosed as severely dynapenic.

### 2.4. Statistical Analysis

The IBM SPSS Version 18 Software suit (SPSS, Chicago, IL, USA) and STATISTICA 13.3 software package (TIBCO Software, Palo Alto, CA, USA) were used to analyze the data collected. The Shapiro–Wilk test was used to assess the distribution of variables. Data were presented as means (M) and standard deviation (SD) for normally distributed continuous variables, as medians (Me) and interquartile range (IQR) for not normally distributed ones and the number of cases and percentage for categorical variables. Proportions were compared using χ^2^ tests or Fisher’s exact test, as appropriate, while the independent samples Student’s *t*-test and the Mann–Whitney U test were used to compare the distribution of continuous variables. It was followed by a multivariable logistic regression including all predictors with a *p*-value less than 0.2 and without significant multicolinearity effect. The variance inflation factor was used to identify correlation between independent variables and the strength of that correlation. Missing values were omitted and statistics in such cases were calculated for the adequately reduced groups. A *p*-value of less than 0.05 was regarded as significant.

### 2.5. Ethics Approval

The source study was approved by the Ethics Committee at the Medical University of Bialystok (no R-I-002/305/2013). All procedures performed in the study were in accordance with the ethical standards of the Medical University of Bialystok research committee and with the Helsinki declaration and its later amendments. The study can be classified as a study of ‘usual practice’. All study participants gave their informed consent to participate in it.

## 3. Results

### 3.1. Study Cohort Characteristics

**Figure 1:**
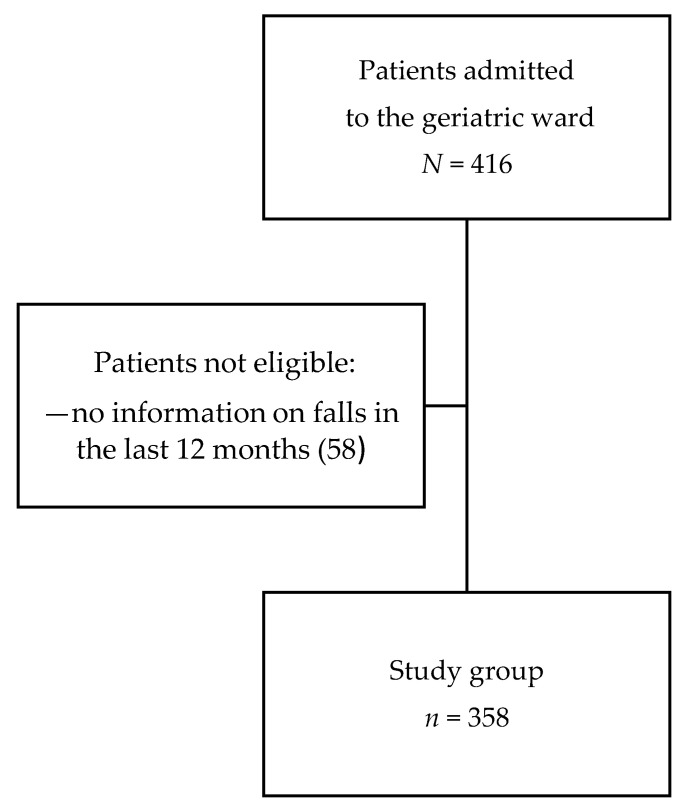
Flow chart of patient enrollment.

A total of 358 (86.1%) patients of 416 hospitalized during the study period—or their guardians—answered the question about the occurrence of falls in the last 12 months and were included in the analysis (Figure 1). Falls in the previous year were reported by 43.9% of them. The median age of patients was 82 years (IQR 77–86) and most of them were above 75 years of age (83.2%) and female (77.9%).

The characteristics of the study groups—fallers and non-fallers—are presented in Table 1 (sociodemographic and health characteristics) and Table 2 (nutritional status and functional characteristics). They did not differ in age, gender and place of residence. The groups differed significantly in the number of chronic diseases (fallers: Me 5.0, IQR 3.0–6.0 versus non-fallers: Me 4.0, IQR 3.0–6.0, *p* = 0.02), but not in the percentage of multimorbidity. The percentage of some diseases was significantly higher in the fallers group: Parkinson’s disease (fallers: 17.8% versus 6.5% in non-fallers, *p* < 0.001), peripheral arterial disease (fallers: 26.8% versus 16.4% in non-fallers, *p* = 0.02) and chronic osteoarthritis (fallers: 82.8% versus 73.6% in non-fallers, *p* = 0.04), but the percentage of dementia, hypertension, ischemic heart disease, history of myocardial infarction, congestive heart failure, TIA/ stroke, diabetes, osteoporosis and chronic kidney disease was not substantially different in both groups. The median number of medications taken, and the percentage of polypharmacy also did not significantly differ. We did not notice any dissimilarities in the frequency of taking any type of medication. Only in the case of quetiapine, the difference was on the verge of statistical significance (fallers: 14.5% versus 8.1% in non-fallers, *p* = 0.06). Median systolic and diastolic blood pressure value at admittance and percentage of orthostatic hypotension were similar in fallers and non-fallers. The groups did not differ in median levels of vitamin B12, serum sodium, serum creatinine, hemoglobin and in mean value of GFR. The percentage of anemia patients was also not significantly different in both groups.

Similar percentages of fallers and non-fallers were hospitalized in the last year (information on the average number of hospitalizations was not available). The Barthel index, the Duke OARS and the MNA-SF scales scores were significantly lower in the fallers’ group, whereas the AMTS and GDS scores were not materially different. Urinary incontinence was reported significantly more frequently in the fallers’ group. The gait speed, the POMA, and the TUG results were significantly worse in the fallers’ group and the percentage of patients with gait speed ≤0.8 m/s was significantly higher (71.0% versus 59.1% in non-fallers, *p* = 0.03). The handgrip strength did not differ significantly between fallers and non-fallers, although such a difference was observed in men. Despite that, no difference was observed in the percentage of patients with dynapenia, as well as those classified as severely dynapenic between fallers and non-fallers. The medium level of frailty status classified with the CFS was significantly higher in fallers (5.0, IQR 4.0–5.0 vs. 4.0, IQR 4.0–5.0 in non-fallers, *p* = 0.03), but the groups did not differ significantly in the percentage of patients assessed as severely frail. The mean BMI as well as percentages of patients with BMI below 24.0 kg/m^2^ and with BMI above 30 kg/m^2^ were not significantly different in fallers and non-fallers. The mean value of serum albumin was notably lower in fallers: 39.0 ± 4.0-g/L vs. 39.5 ± 4.0-g/L in non-fallers, *p* = 0.03, but the groups did not differ significantly in the percentages of patients at risk of malnutrition according to the MNA-SF score.

### 3.2. Independent Predictors of Being a Faller

A direct multivariable logistic regression analysis was carried out on faller status as outcome and 21 predictors: age 75+, multimorbidity, cardiac heart failure, peripheral arterial disease, history of stroke/TIA, Parkinson’s disease, chronic osteoarthritis, POMA, Barthel Index, IADL score, gait speed, CFS status, urinary incontinence, MNA-SF score, albumin value, vitamin B12 level and taking certain medications (quetiapine, vitamin D, diuretics, BDA and SSRI) (Table 3). A few variables meeting the criterion *p* < 0.2 (number of chronic diseases, gait speed ≤ 0.8 m/s and severe frailty) were not included in the logistic regression after testing for correlation with other variables and for their multicollinearity effect. The handgrip variable was introduced into the regression model, although it did not meet criteria (*p* = 0.22) because when it was separated by sex, it did it. Significantly higher odds for faller status was observed only for Parkinson’s disease (odds ratio, 2.82; 95% CI, 1.07–7.45; *p* = 0.04) and osteoarthritis (odds ratio, 2.08; 95% CI, 1.02–4.23; *p* = 0.04).

A test of this model against a constant-only model was statistically reliable, χ (21, *N* = 265) = 32.90, *p* < 0.047, indicating that the characteristics, as a set, reliably distinguished between fallers and non-fallers. An overall prediction success rate in logistic regression constituted 64.2%, with 60.2% of fallers and 67.9% of non-fallers predicted correctly.

### 3.3. Study Participants and Nonparticipants

Patients hospitalized in the geriatric ward during the study period, but excluded from the analysis because of the missing data on falls in the previous 12 months did not differ from study participants in age, gender and in a number of characteristics, but were more often severely frail, demented, and had significantly worse nutritional and functional parameters (Table 4).

## 4. Discussion

Our study has confirmed that falls are a common problem among geriatric ward patients. 40% of patients in the study group reported falls in the previous year. The percentage of pre-hospitalization falls in our study was similar to that observed by Van Ancum et al. in a study conducted on patients of 70 years of age and older admitted to the academic teaching hospital in Amsterdam [4]. The unfavorable profile of health problems in our patients certainly contributed to this outcome. A high prevalence of such recognized risk factors for falls as Parkinson’s disease, osteoarthritis, urinary incontinence, peripheral arterial disease and dependency on others in basic and instrumental activities of daily living was observed in our study participants. Characteristics of gait and balance (gait speed, the POMA score or the TUG score) were significantly worse in the fallers’ group, who were also frailer according to the CFS scale.

No significant association with being a faller was observed in our study for many other recognized risk factors for falls. Various studies confirmed, for instance, that more advanced age, female gender, multimorbidity, polypharmacy or taking specific types of medications (for instance neuroleptics, diuretics or antihypertensive medications) increased the risk of falls, but it was not observed in our study [11,12,33,34]. We did not note any association between falls and orthostatic hypotension although it was also a well-known risk factor of falls [7]. This fact may have resulted from a retrospective assessment of the occurrence of falls. However, results of other published studies on various risk factors of falls are also slightly contradictory. According to a number of authors, for instance, the use of two or more fall risk-increasing drugs, rather than polypharmacy, is an independent risk factor for falls [35].

Several studies suggested that the nutritional status and BMI should be evaluated when assessing the risk for falls in older age. Systematic review and meta-analysis confirmed a *U*-shaped association observed between BMI and the risk for falls, with the nadir between 24.5 and 30 kg/m^2^ [6]. We did not observe this association in our study. The group of fallers had significantly lower scores of MNA-SF and lower levels of albumin, suggesting an association between falls and malnutrition in our study group. Nevertheless, it is certainly worth examining the relationship between falls and nutritional status in geriatric patients in a prospective study, as nutritional biomarkers assessed in the retrospective projection of the study may not necessarily be sensitive enough to properly assess their relationship to falls.

Parkinson’s disease was the most significant variable independently associated with falls in our research. It corresponded with the results achieved by other authors [36]. The most important factors related to falls in people with Parkinson’s disease comprise of failure of the autonomic nervous system combined with orthostatic hypotension and adverse effects of medications used [37]. Osteoarthritis was another significant variable independently associated with falls in our study and it was consistent with other authors’ observations [38].

Contrary to our expectations and to the results of a number of studies, [13] our research did not confirm that dynapenia (suggestive for sarcopenia) was a statistically significant variable associated independently with falls experienced over the last year in patients admitted to the geriatric ward. “Fallers” and “non-fallers” did not differ in the prevalence of dynapenia and severe dynapenia (defined as dynapenia combined with gait speed≤ 0.8 m/s and/or TUG time ≥ 20 s). Sarcopenia prevalence differs depending on the population assessed and sarcopenia definition applied. The recently published systematic review and meta-analysis confirmed that older sarcopenic individuals had a significantly higher risk of falls compared with non-sarcopenic individuals, independently of study design, population, sex, sarcopenia definition, continent and study quality [13]. However, several researchers indicate that this analysis may not include important studies in which such a relationship could not be confirmed [39], for example, a study by Henwood et al. conducted in nursing home residents [40] and a study by Schaap et al. conducted in older participants of the Longitudinal Aging Study Amsterdam [41]. Moreover, according to a study by Sim et al. assessing the association between sarcopenia diagnosed with four different criteria and falls-related hospitalization risk in the cohort of community-dwelling older Australian women, sarcopenia did not increase the relative hazard ratio for falls-related hospitalization before or after adjustment for age [42]. Unfortunately, many of these assessments did not take into account the independent, simultaneous impact of other determinants of falls significant in older patients, although—as mentioned above—they were multifactorial in nature. Due to the observed tendency of not taking history of falls in the previous year in very frail, functionally dependent and bedridden individuals admitted to the department, it is necessary to consider the possibility of the underestimation of dynapenia/sarcopenia influence on the faller status in our study. Furthermore, sarcopenia and malnutrition are phenomena evolving over time, the risk factors of which are often derived from childhood. These include, for example, a lack of physical activity that contributes to a number of chronic diseases and adverse sequelae [43]. The cross-sectional plan of our study, without data on sarcopenia assessed prior to admission, could also mean that we do not find a correlation between low muscle mass and muscle capacity and falls.

Albeit the assessment of the risk of falls is an important element of the comprehensive geriatric assessment and the question on falls in the last 12 months should be asked at the beginning of the screening, as many as 13.9% of patients hospitalized in the study period or their guardian, were not asked about or were unable to answer this question. This fact could have been caused by the possible assumption that patients with significantly reduced mobility did not experience falls although the results of a number of studies contradicted this presupposition [44]. A similar tendency was observed in case of the active standing test for detection of orthostatic hypotension [20]. Additionally, in a large number of patients, the hand grip strength, gait speed and TUG test were not assessed (as indicated in Table 1), which could also have influenced the results of the study.

The study has got some limitations that ought to be emphasized. First of all, because of the temporal relationship of fall incidence with baseline medical information, we can say that the study identified potential factors associated with falls, but not actual “predictors” of falls, and the word “predictor” should be treated as a mathematical concept used in regression analysis, rather than as a factor determining the occurrence of a phenomenon. Additionally, as it was underlined above, the study was conducted not in a random sample from the general population, but in a convenient sample of patients admitted to the geriatric ward, therefore, the results can be generalized for patients of similar settings only. Prospective population-based studies should be conducted to explore our findings more thoroughly and overcome these limitations. As our study was based on the secondary analysis of data previously collected, some pieces of information were limited, as indicated in tables. Moreover, the nonparticipant group differed significantly from the study group in various important characteristics. This could also have had an influence on the final results.

## 5. Conclusions

In summary, the study revealed that falls were a common problem among geriatric ward patients and affected up to 43.9% of them. Parkinson’s disease and osteoarthritis were the main independent predictors connected with the significantly higher odds for being a faller before admittance to the hospital. Sarcopenia and some other previously confirmed risk factors for falling were not connected with a faller status in geriatric ward patients, although their influence may be underestimated due to the tendency of not taking the history of falls experienced in very frail, functionally dependent and bedridden individuals.

## Figures and Tables

**Table 1:** Characteristics of study participants—sociodemographic and health correlates of falls.

Characteristic	All	Fallers	Non-Fallers	*p* ^1^	Missing Values
*n* (%)	358 (100.0)	157 (43.9)	201 (56.15)		
Age (y), Me (IQR)	82 (76–86)	82 (77–86)	82 (76–85)	0.23	–
Age (75+), *n* (%)	298 (83.2)	136 (86.6)	162 (80.6)	0.13	–
Sex (F), *n* (%)	279 (77.9)	123 (78.3)	156 (77.6)	0.86	–
Residence (rural), *n* (%)	68 (19.0)	28 (17.8)	40 (19.9)	0.68	–
Number of chronic diseases, Me (IQR)	5.0 (3.0–6.0)	5.0 (3.0–6.0)	4.0 (3.0–6.0)	0.02	–
Multimorbidity, *n* (%)	200 (55.9)	96 (26.8)	104 (51.7)	0.07	–
Number of drugs, Me (IQR)	7.0 (5.0–9.0)	7.0 (5.5–9.5)	7.0 (5.0–9.0)	0.25	8
Polypharmacy, *n* (%)	281 (80.3)	126 (82.9)	155 (78.3)	0.34	8
Hospitalization in the last year, *n* (%)	102 (28.6)	45 (28.7)	57 (28.5)	0.97	1
Chronic diseases					
Parkinson’s disease, *n* (%)	41 (11.5)	28 (17.8)	13 (6.5)	<0.001	–
Dementia, *n* (%)	102 (28.5)	48 (30.1)	54 (26.9)	0.44	–
Hypertension, *n* (%)	288 (80.5)	128 (81.5)	160 (79.6)	0.64	–
Ischemic heart disease, *n* (%)	195 (54.5)	87 (55.4)	108 (53.7)	0.75	–
Myocardial infarction, *n* (%)	36 (10.0)	17 (10.8)	19 (9.5)	0.66	–
Atrial fibrillation, *n* (%)	82 (22.9)	36 (22.9)	46 (22.9)	0.99	–
Chronic cardiac failure, *n* (%)	135 (37.7)	66 (42)	69 (34.3)	0.14	–
NYHA class I/II, *n* (%)	67 (18.7)	32 (20.4)	35 (17.4)		
NYHA class III/IV, *n* (%)	68 (19)	34 (21.7)	34 (16.9)		
Peripheral arterial disease, *n* (%)	75 (20.1)	42 ( 26.8)	33 (16.4)	0.02	–
Stroke/ TIA, *n* (%)	41 (11.5)	23 (14.7)	18 (9.0)	0.09	–
Osteoarthritis, *n* (%)	278 (77.7)	130 (82.8)	148 (73.6)	0.04	–
Osteoporosis, *n* (%)	65 (18.2)	27 (17.2)	38 (18.9)	0.68	–
Diabetes, *n* (%)	104 (21.9)	50 (31.9)	54 (26.9)	0.30	–
Chronic kidney disease, *n* (%)	186 (52.0)	82 (52.2)	104 (51.7)	0.93	–
Orthostatic hypotension, *n* (%)	51 (15.7)	25 (16.6)	26 (15.0)	0.70	34
Vitamin B12, pg/mL, Me (IQR)	332.6 (242.2–430.2)	331.3 (222.1–422.2)	337.30 (256.9–431.6)	0.10	35
Na, mmol/L, Me (IQR)	140.0 (138.0–141.0)	140.0 (138.0–141.0)	140.0 (138.0–141.0)	0.94	10
eGFR, mL/min/1.73 m^2^, M (SD)	58.3 (16.7)	58.2 (16.8)	58.4 (16.7)	0.91	10
Serum creatinine, mmol/L, Me (IQR)	86.6 (74.3–105.2)	85.75 (72.5–105.2)	87.52 (76.0–103.4)	0.76	10
Hemoglobin, mmol/L, Me (IQR)	7.8 (7.2–8.5)	7.9 (7.2–8.4)	7.93 (7.2–8.5)	0.83	11
Anemia, *n* (%)	149(42.9)	72 (46.2)	77 (40.3)	0.27	11
Medications					
α1-blockers, *n* (%)	21 (6.0)	8 (5.3)	13 (6.6)	0.60	9
Thyroid hormones, *n* (%)	26 (7.5)	11 (7.2)	15 (7.6)	0.89	9
Antithyroid drugs, *n* (%)	2 (06)	–	2 (1.0)	0.51	9
Quetiapine, *n* (%)	38 (10.9)	22 (14.5)	16 (8.1)	0.06	9
Neuroleptics, *n* (%)	49 (14.0)	25 (16.5)	24 (12.2)	0.26	9
Vitamin D, *n* (%)	79 (22.6)	29 (19.0)	50 (25.4)	0.16	9
ACE-Is/ ARBs, *n* (%)	231 (66.2)	95 (62.5)	136 (69.0)	0.21	9
Memantine, *n* (%)	11 (3.2)	3 (2.0)	8 (4.0)	0.27	9
AChE-Is, *n* (%)	35 (10.0)	17(11.2)	18 (9.1)	0.53	9
ß-blockers, *n* (%)	222 (63.6)	98 (64.5)	124 (63.0)	0.77	9
Calcium channel blockers, *n* (%)	102 (29.2)	46 (30.3)	56 (28.4)	0.70	9
Digoxin, *n* (%)	24 (6.9)	13 (8.6)	11 (5.6)	0.28	9
Diuretics, *n* (%)	165 (47.3)	65 (42.8)	100 (50.8)	0.14	9
Lipid lowering drugs, *n* (%)	125 (35.8)	51 (33.6)	74 (37.6)	0.44	9
BDA, *n* (%)	40 (11.4)	22 (14.4)	18 (9.1)	0.13	8
SSRI, *n* (%)	98 (28.0)	49 (32.2)	49 (24.9)	0.13	9
NSAIDs, *n* (%)	24 (6.9)	12 (7.9)	12 (6.1)	0.53	9

^1^—χ^2^ test or Fisher’s exact test, as appropriate, for categorical variables; *t*-test for independent samples or Mann–Whitney test for continuous or interval variables. In all analyses a two-tailed *P*-value of less than 0.05 was regarded as significant. ACE-Is—angiotensin converting enzyme inhibitors; AChE-I—acetylcholinesterase inhibitors; ARBs—angiotensin II receptor blockers; BDA—benzodiazepines; BP—blood pressure—eGFR—glomerular filtration rate; IQR—interquartile range; M—mean value; Me—median value; *n*—number of cases; Na—serum sodium; NSAIDs—non-steroidal anti-inflammatory drugs; SSRI—selective serotonin reuptake inhibitors; SD—standard deviation; TIA—transient ischemic attack.

**Table 2:** Characteristics of study participants—functional and nutritional correlates of falls.

Characteristic	All	Fallers	Non-Fallers	*p* ^1^	Missing Values
*n* (%)	358 (100.0)	157 (43.9)	201 (56.15)		
Barthel index, Me (IQR)	95 (80–100)	90 (75–95)	95 (85–100)	0.004	6
IADL, Me (IQR)	8.0 (5.0–11.0)	7.0 (4.0–10.5)	9.0 (6.0–12.0)	0.01	10
AMTS, Me (IQR)	8.0 (6.0–9.0)	8.0 (7.0–9.0)	8.0 (6.0–9.0)	0.58	21
GDS, Me (IQR)	6.0 (3.0–10.0)	7.0 (3.0–10.0)	6.0 (3.0–9.0)	0.34	34
Handgrip, kg, Me (IQR)	18.4 (14.1–23.0)	18.1 (13.9–22.3)	18.8 (14.3–24.3)	0.22	32
men, kg, Me (IQR)	26 (21.0–32.3)	26.1 (21.5–29.6)	30.0 (23.0–35.5)	0.047	12
women, kg, Me (IQR)	16.7 (12.9–20.5)	16.8 (12.7–19.9)	17.3 (13.5–21.3)	0.2	20
Dynapenia, *n* (%)	143 (43.9)	73 (47.4)	70 (40.7)	0.26	32
Severe dynapenia, *n* (%)	95 (32.8)	48 (36.4)	47 (29.7)	0.26	68
Gait speed, m/s, Me (IQR)	0.65 (0.40–0.95)	0.60 (0.37–0.86)	0.71 (0.43–1.05)	0.01	49
Gait speed ≤ 0.8 m/s, *n* (%)	200 (64.7)	103 (71.0)	97 (59.1)	0.03	49
POMA, Me (IQR)	23.0 (18.0–28.0)	22.0 (17.0–28.0)	25.0 (19.0–28.0)	0.005	49
TUG, s, Me (IQR)	17.1 (11.7–27.2)	17.4 (13.0–28.3)	16.5 (11.3–25.2)	0.25	64
TUG ≥ 20 s, *n* (%)	121 (41.2)	55 (41.7)	66 (40.7)	0.91	64
CFS, Me (IQR)	4.0 (4.0–5.0)	5.0 (4.0–5.0)	4.0 (4.0–5.0)	0.03	–
Severe frailty, *n* (%)	58 (16.2)	31 (19.7)	27 (13.4)	0.11	–
Urinary incontinence, *n* (%)	146 (41.4)	78 (49.7)	68 (34.7)	0.004	5
BMI, kg/m2, M (SD)	29.35 (5.99)	29.33 (5.97)	29.37 (6.02)	0.92	34
BMI < 24 kg/m^2^, *n* (%)	59 (18.2)	26 (18.7)	33 (17.9)	0.84	34
BMI > 30 kg/m^2^, *n* (%)	136 (42.0)	57 (41.0)	79 (42.7)	0.76	34
MAC, cm, M (SD)	28.2 (4.0)	28.1 (4.0)	28.3 (3.9)	0.82	47
MAC ≤ 22 cm, *n* (%)	73 (23.5)	35 (23.8)	38 (23.2)	0.89	47
CC, cm, M (SD)	34.8 (4.5)	34.6 (4.7)	35.0(4.4)	0.50	47
CC < 31 cm, *n* (%)	53 (17.0)	29 (19.7)	24 (14.6)	0.23	47
Albumin, g/L, M (SD)	39.3 (3.7)	39.0 (4.0)	39.5 (4.0)	0.03	24
MNA-SF, Me (IQR)	12.0 (10.0–13.0)	12.0 (9.0–13.0)	13.0 (10.0–14.0)	0.04	10
MNA-SF score < 8, *n* (%)	46 (13.2)	21 (13.6)	25 (12.9)	0.84	10

^1^—χ^2^ test or Fisher’s exact test, as appropriate, for categorical variables; *t*-test for independent samples or Mann–Whitney test for continuous or interval variables. In all analyses a two-tailed *P*-value of less than 0.05 was regarded as significant. AMTS—Abbreviated Mental Test Score; BMI—body mass index; CC—calf circumference; CFS—7-point Clinical Frailty Scale; IADL—instrumental activities of daily living; IQR—interquartile range; M—mean value; Me—median value; MAC—mid arm circumference; MNA-SF— Mini Nutritional Assessment Short Form; *n*—number of cases; POMA—Performance Oriented Mobility Assessment; TUG—Timed Up-and-Go test; SD—standard deviation.

**Table 3:** Results of the direct logistic regression model indicating characteristics associated with falls.

Predictors	OR (95% CI)	*p*
Age, 75+ years	1.17 (0.54–2.53)	0.69
Multimorbidity	1.01 (0.52–1.98)	0.97
Chronic cardiac failure	1.67 (0.88–3.14)	0.12
Peripheral arterial disease	0.90 (0.41–2.00)	0.80
Stroke/TIA	1.82 (0.71–4.67)	0.22
Parkinson’s disease	2.82 (1.07–7.45)	**0.04**
Osteoarthritis	2.08 (1.02–4.23)	**0.04**
POMA	0.95 (0.87–1.02)	0.16
Barthel index	1.01 (0.98–1.03)	0.53
IADL	0.99 (0.89–1.12)	0.96
Gait speed	0.78 (0.18–3.39)	0.69
CFS	0.76(0.50–1.16)	0.21
Urinary incontinence	1.37 (0.75–2.49)	0.31
MNA-SF	1.02 (0.91–1.15)	0.73
Albumin	1.00 (0.92–1.09)	0.96
Vitamin B12	0.99 (0.99–1.00)	0.12
Quetiapine	1.66 (0.64–4.31)	0.30
Vitamin D	1.08 (0.54–2.17)	0.82
Diuretics	0.69 (0.39–1.21)	0.19
BDA	1.29 (0.54–3.08)	0.56
SSRI	1.30 (0.69–2.46)	0.42

Bold values indicate statistical significance at the *p* < 0.05 level. BDA—benzodiazepines; CFS—Clinical Frailty Scale; CI—confidence interval; IADL—instrumental activities of daily living; MNA-SF—Mini Nutritional Assessment Short Form; OR—odds ratio; POMA—Performance Oriented Mobility Assessment—SSRI—selective serotonin reuptake inhibitor; TIA—transient ischemic attack.

**Table 4:** Characteristics of participants and nonparticipants of the study.

Parameter	Participants	Nonparticipants	*p* ^1^
No. (%) of patients	358 (86.1)	58 (13.9)	
Age, years, Me (IQR)	84 (78.8–87.3)	82 (76–86)	0.22
Age, 75+, *n* (%)	298 (83.2)	52 (89.7)	0.22
Gender, women, *n* (%)	279 (77.9)	43 (74.1)	0.52
Barthel index, Me (IQR)	95 (80–100)	42.5 (15–70)	<0.001
IADL, Me (IQR)	8 (5.0–11.0)	0.0 (0.0–4.0)	<0.001
POMA, Me (IQR)	23 (18–28)	8.0 (8.0–15.0)	<0.001
CFS, Me (IQR)	4.0 (4.0–5.0)	6.0 (6.0–7.0)	<0.001
Not able to walk, *n*(%)	18 (5.2)	25 (47.2)	<0.001
Severe frailty, *n* (%)	58 (16.2)	44 (75.9)	<0.001
Sarcopenia, *n* (%)	143 (43.9)	21 (87.5)	<0.001
Urinary incontinence, *n* (%)	146 (41.4)	47 (81.0)	<0.001
Dementia, *n* (%)	102 (28.5)	31 (53.4)	<0.001
AMTS, Me (IQR)	8 (6.0–9.0)	6.5 (3.0–8.75)	<0.001
Neuroleptics, *n* (%)	49 (14.0)	18 (31.6)	0.001
Parkinson’s disease, *n* (%)	41 (11.5)	14 (24.1)	0.008
TIA/stroke, *n* (%)	41 (11.5)	15 (25.9)	0.003
MNA-SF<8, *n* (%)	46 (13.2)	26 (46.4)	<0.001
Albumin, g/L, M (SD)	36.6 (3.6)	39.3 (3.7)	<0.001

^1^—χ^2^ test for categorical variables; *t*-test or Mann–Whitney test for continuous or interval variables. In all analyses a two-tailed *P*-value of less than 0.05 was regarded as significant. AMTS—Abbreviated Mental Test Score; CC—calf circumference; CFS—7-point Clinical Frailty Scale; IADL—instrumental activities of daily living; IQR—interquartile range; M—mean value; Me—median value; MNA-SF—Mini Nutritional Assessment Short Form; n—number of cases; POMA—Performance Oriented Mobility Assessment; SD—standard deviation; TIA—transient ischemic attack.

## Abbreviations

ACE-I	angiotensin converting enzyme inhibitor
AChE-I	acetylcholine esterase inhibitor
AMTS	abbreviated mental test score
ARB	angiotensin receptor blocker
BDA	benzodiazepines
BMI	body mass index
CFS	Clinical Frailty Scale
CKD-EPI	Chronic Kidney Disease Epidemiology Collaboration
CI	confidence interval
GDS	Geriatric Depression Scale
GFR	glomerular filtration rate
IADL	instrumental activities of daily living
IQR	interquartile range
KDOQI	Kidney Disease Outcome Quality Initiative
M	mean
Me	median
MNA-SF	Mini Nutritional Assessment Short Form
NSAIDs	non-steroidal anti-inflammatory drugs
NYHA	New York Heart Association
OARS	Older Americans Resources and Services
OR	odds ratio
POMA	Performance Oriented Mobility Assessment
SD	standard deviation
SSRI	selective serotonin reuptake inhibitors
TIA	transient ischemic attack
TUG	Timed Up-and-Go Test
